# On the interaction of speakers’ voice quality, ambient noise and task complexity with children’s listening comprehension and cognition

**DOI:** 10.3389/fpsyg.2015.00871

**Published:** 2015-06-24

**Authors:** Viveka Lyberg-Åhlander, K. J. Brännström, Birgitta S. Sahlén

**Affiliations:** Department of Clinical Sciences, Logopedics, Phoniatrics and Audiology, Lund UniversityLund, Sweden

**Keywords:** comprehension, voice, noise, cognition, children

## Abstract

Suboptimal listening conditions interfere with listeners’ on-line comprehension. A degraded source signal, noise that interferes with sound transmission, and/or listeners’ cognitive or linguistic limitations are examples of adverse listening conditions. Few studies have explored the interaction of these factors in pediatric populations. Yet, they represent an increasing challenge in educational settings. We will in the following report on our research and address the effect of adverse listening conditions pertaining to speakers’ voices, background noise, and children’s cognitive capacity on listening comprehension. Results from our studies clearly indicate that children risk underachieving both in formal assessments and in noisy class-rooms when an examiner or teacher speaks with a hoarse (dysphonic) voice. This seems particularly true when task complexity is low or when a child is approaching her/his limits of mastering a comprehension task.

## Background

Poor listening environments are challenging for typically developing children with normal hearing and even more so for children struggling with listening comprehension in different disability groups (Khalfa et al., 2004). Noise that interferes with sound transmission, forces students to allocate cognitive capacity to suppress the task irrelevant input. This allocation spares less capacity for the processing and recall of the content (Shield and Dockrell, 2008; Sörqvist, 2010; Klatte et al., 2013). However, little attention has been paid to the role which source signal alterations, for example changes in speakers’ speaking rate, may play for children’s listening comprehension. In one of our studies, 8-year-olds listened to recorded sentences read aloud by a speech language pathologist speaking with either fast, normal or slow speech rate (Haake et al., 2014). The slower speech rate was generally associated with better performance on a language comprehension test. Children with stronger working memory capacity (WMC) benefitted more from slow speech rate than their peers, but only for more complex sentences. The slower speech rate did not improve performance on the more complex tasks in children with weaker WMC, probably because these tasks were beyond their grasp. It was concluded by the authors that it is when the child is just about to master a comprehension task that slower speech is beneficial.

## The Influence of Adverse Voice Quality on Listening Comprehension

Alterations of speech rate may degrade the source signal but the risk for degradation is higher when a speaker speaks with dysphonic voice or a non-native accent (Mattys et al., 2012). A dysphonic (coml. hoarse) voice is defined as a voice that qualitatively may deviate from the ‘typical’ in a number of ways, e.g., pressed (hyperfunctional), breathy, rough and/or instable. The cause is an organically or functionally impaired voice function. Only a couple of studies have investigated the impact of voice quality on listening comprehension (Morton and Watson, 2001; Rogerson and Dodd, 2005). In spite of small differences in methodology, the authors’ conclusions are convergent: a dysphonic teacher-voice hampers children’s comprehension and listeners may judge dysphonic voices more negatively than typical voices with possible effects on motivation and learning (Morton and Watson, 2001).

Our own studies corroborate these findings and extend existing knowledge in some explorative and experimental studies. More specifically, we studied the impact of teachers’ voice quality on children’s accuracy, reaction times in a listening comprehension task with increasing complexity. We further studied the children’s subjective experience of the voice. The experiments were performed either in silence or in background babble-noise (Brännström et al., 2014; Lyberg-Åhlander et al., 2015a,b).

We used a digitalized version of a language comprehension test, the TROG-2 (Bishop, 2003, 2009), which is a picture selection test consisting of 80 sentences, organized into 20 blocks with increasing lingusitic complexity. Accuracy, self-corrections and speed (response times) were measured. To assess WMC, the Competing Language Processing Task (CLPT; Gaulin and Campbell, 1994), was used. The CLPT is a test used for assessment of complex WMC. In the CLPT, initially the participant is asked to judge the semantic acceptability of a sentence and thereafter, in blocks of 1–6 sentences (a total of 42 sentences), they are asked to repeat the final words of each sentence. To assess executive functioning the Elithorn’s Mazes (EM, WISC–IV; Wechsler, 2004) were used. In all four studies reported below, we utilized a between-group design. The children listened to the recorded sentences read by the same female speaker, either using her normal voice or a dysphonic voice, either mimicked or induced through vocal loading. In each study, around 90 typically developing normal hearing 8-year olds from schools in Southern Sweden were included.

The first study by Lyberg-Åhlander et al. (2015a) was performed with a mimicked dysphonic voice and no ambient noise. We found no overall effect of the mimicked and moderately dysphonic voice on comprehension. However, the children listening to the dysphonic voice achieved significantly lower TROG-2 scores for sentences in the more complex blocks of the test (“the man but not the horse is jumping”). These children also made significantly more self-corrections than those listening to the typical voice, but this was restricted to the less complex sentences (“the girl is sitting”). Decreased accuracy in more complex tasks was interpreted as indicating that the mimicked dysphonic speaker’s voice forced children to allocate capacity to the processing of the voice signal at the expense of listening comprehension, particularly when the linguistic difficulty is of borderline complexity for the child. The scores on EM correlated significantly to the TROG-2 results. We also analyzed response times. Response time is often used as measures for listening effort in adults and are, by some researchers, considered a reliable measure for listening effort in children (Hick and Tharpe, 2002). Preliminary analyses yielded no overall difference between voice qualities, but response times increased with task difficulty in both conditions and were longer for girls in the dysphonic condition (with mimicked and vocally loading induced dysphonia) as compared to the girls in the typical voice condition and to the boys in both conditions. Based on our data we believe that several other factors such as interest, motivation, and socio-cultural aspects underpin response times.

## The Combined Effect of a Dysphonic Voice Quality and Noise on Comprehension

In yet another study, Lyberg-Åhlander et al. (2015b) explored what happens when children listen to a typical versus a dysphonic speaker in simultaneous background babble-noise. Speaking in a noisy environment will also change the voice quality of a speaker with a typical voice. Therefore, the voice-paradigm had to be altered to achieve two ecologically valid voice qualities. The female speaker was now recorded as she was making herself heard while speaking in babble-noise. During the study, one group of children listened to the speaker recorded with her somewhat strained but ‘typical’ voice in babble-noise (Holube et al., 2010) and another group listened to her dysphonic voice, which was induced by a vocal loading task before the recording. The vocal loading task refers to when the speaker was asked to read out loud for 30 min in 85dB babble-noise (Whitling et al., 2015). This mode of vocal loading, common in noisy classrooms, often causes a speaker with a healthy voice to raise the fundamental frequency and to use a more hyperfunctional phonation. Speaking over noise changes the spectrum of the voice as compared to the typical voice, and may result in an increase or decrease of noise in the higher part of the spectrum. The ecological validity of the voice qualities (typical/dysphonic) was assessed by an expert panel where the dysphonic voice was judged as significantly more disordered.

The TROG-2 results did not differ between the groups. We concluded that the background babble-noise, present in both conditions, might have masked a possible additional effect of the dysphonic voice. However, significant differences between voice conditions were found for the interaction between WMC and linguistic task-complexity, particularly in tasks representing intermediate difficulty. In the dysphonic voice condition, children with stronger WMC scored significantly higher on easier blocks, whereas, in the typical voice condition the cognitively stronger children scored higher on more difficult blocks.

Unfortunately, a direct comparison between the results of these two studies Lyberg-Åhlander et al. (2015a,b), is impeded by differences in transducers used to present the voices and by the use of mimicked versus authentic dysphonia. Therefore, the relative contribution of the voice quality *per se* cannot be teased out. Even so, importantly, these combined results indicate synergistic detrimental effects on children’s listening comprehension in a class-room when dysphonic teachers try to make themselves heard in ambient noise.

## The Interaction of Perceptual Load, Task Complexity and Attitude to Voice

Some of the results from these studies are complex and at first counterintuitive. For instance, why should a dysphonic voice lead children to make more self-corrections on easier tasks than on more difficult tasks? According to the perceptual load theory (Lavie, 2005), sufficiently easy tasks free cognitive capacity to process task-irrelevant stimuli in adults. This may explain the increased amount of self-corrections in the easier tasks in the dysphonic condition in the earlier study (Lyberg-Åhlander et al., 2015a). The children may have had the cognitive capacity needed to process, or even to get disturbed by, the dysphonic voice. Results in the later study by Lyberg-Åhlander et al. (2015b) may be explained accordingly. In this study, children with stronger WMC, performed better on the more difficult tasks when listening to the typical voice in noise (i.e., lower perceptual load and higher cognitive complexity) and on the easier tasks when listening to the dysphonic voice in noise (i.e., higher perceptual load and lower cognitive complexity). Detrimental effects of adverse conditions on listening comprehension may thus decrease when perceptual load increases, as was the case for children with stronger WMC. This is in line with the perceptual load hypothesis stating that, in adults, the effect of task-irrelevant stimuli diminishes when the task itself is sufficiently complex.

It has previously been suggested that negative attitudes toward a teacher’s voice may influence the teacher–child relation and as a consequence may influence motivation and learning outcomes negatively (Morton and Watson, 2001). In Brännström et al. (2014), we therefore investigated children’s subjective ratings of the speakers’ voices using data from Lyberg-Åhlander et al. (2015b). Children thus listened to the same speaker using typical voice or with vocal-loading induced dysphonic voice in ambient babble-noise. Self-reports from the children of perceived effort and attitude to the teacher voice were collected after the listening comprehension task. The children’s judgments were collected with the help of emoticons, later transformed to a five-step Lickert scale. The dysphonic voice, as expected, received lower ratings compared to the ratings of the typical voice. Example children’s opinions were that the speaker with the dysphonic voice was ‘stressed’ or ‘nice but determined.’ Children in the typical voice group who made more positive ratings of the voice, performed better on earlier items in the TROG-2. Accordingly, the perception of the voice related to the child’s performance for low complexity tasks. Self-assessments in a pediatric population are problematic for a range of reasons and further studies are needed. Children may rate both their own and other’s behavior in relation to their self-efficacy, to their own task performance and to other contextual circumstances, especially when made in hind-sight. Children might also try to either deceive or please the test-leader (DeRight and Carone, 2015).

## A Developmental Perspective on Human Voice Recognition

During adverse listening conditions, whether the origin is related to the speaker, the environment or the listener, compensatory mechanisms emerge, and recalibration takes place in ‘the human speech recognizer.’ Memory representations of talkers’ voices are stored in long-term memory (Mattys et al., 2012). A developmental perspective of this type of perceptual learning in talker recognition has been proposed by Creel and Jimenez (2012). According to these authors, young preschool children, with typical cognitive and linguistic development will cease to filter out acoustical cues during development and successively internalize such cues and finally become efficient at talker recognition and understanding as adults. This developmental perspective suggests that differences in adaptation to speakers’ voice quality could be related to the child’s cognitive capacity. With our between-group we can only speculate that the children with a stronger cognitive capacity and better listening comprehension may have developed more stable talker-templates. They would perhaps, as a result, be less disturbed than cognitively less mature children by a mismatch between a degraded talker signal (such as when their teacher suddenly becomes dysphonic), and their memory representations of the speaker’s ‘normal’ voice.

## Implications for Future Studies

We have recently taken several steps to reach higher ecological validity in on-going studies. As for the interaction of noise and voice, in Lyberg-Åhlander et al. (2015b), we aimed to simulate an actual classroom situation by using multi-talker babble International Speech Test Signal (ISTS; Holube et al., 2010), with six female voices constituting the noise source. Our choice of speaker and babble-noise was inspired by Zekveld et al. (2014), who conclude that speech recognition is more influenced when the disturbing signal is produced by a person of the same gender as the speaker of the target signal and, that the cognitive load is greater. This is especially true when the disturbing signal is derived from a source that is spatially close to the target signal. Our choice of a non-semantic babble-noise may, however, have made the comprehension task somewhat easier compared to if the babble would have been possible to understand (Rosen et al., 2013). Further studies will therefore utilize semantic babble.

In current studies we are addressing effects of suboptimal listening conditions on long-term memory. It is possible that the influence of voice quality on performance and attitude will change if children are assessed after a period of time when long-term memory integration has occurred. Thus a measurement that is not restricted to comprehension of sentences but that includes also comprehension of narratives both in direct connection to the task and after a period of time, could investigate the effects of episodic memory. Further, multimodal aspects of comprehension and memory in adverse conditions are explored by the use of a virtual teacher agent. This enables the systematic study of visual versus audio-visual aspects of comprehension. Using a mixture of techniques (optical markers and infrared 3D-gitter, Dutta, 2012; Gonzalez-Jorge et al., 2013) we can record both macro- (postures, gestures) and micro-level (eye blinks and lips) movements and map them onto a digital 3D-character. A virtual teacher allows further experimental control of visual aspects (sex/gender, age, clothing, etc.) as well as postural movement and gestures (amplitude, velocity, synchronization, etc.) in combination with controlled voice recordings.

## Conclusion

Today, assessment and intervention in children with language, hearing, and/or cognitive impairments are increasingly based on knowledge of how cognitive functioning and acoustic processing interact. There is, however, an apparent lack of knowledge on how noise interacts with these factors. Environmental noise not only influences children’s comprehension but also teacher’s voices. Voice problems reach a point-prevalence of thirteen percent in Swedish teachers (Lyberg-Åhlander et al., 2011) and a career prevalence close to 60% (Roy et al., 2004). The summary of our results indicates that children risk underachievement in both formal assessments and in noisy class-rooms if an examiner or teacher speaks with a dysphonic voice, particularly when tasks demands are too low or when the child is approaching her/his limits of mastering a comprehension task. Our studies indicate that individual variations in cognitive capacity must be taken into consideration in research on the interaction of task complexity and on adverse listening conditions pertaining to the speaker and the noise environment.

## Conflict of Interest Statement

The authors declare that the research was conducted in the absence of any commercial or financial relationships that could be construed as a potential conflict of interest.
